# Exploring perspectives on chronic obstructive pulmonary disease in people who smoke heroin: a qualitative study

**DOI:** 10.3399/bjgpopen20X101055

**Published:** 2020-07-15

**Authors:** Rebecca Nightingale, Paul Griffiths, Kevin Mortimer, Paul Walker, Tara Byrne, Kerry Marwood, Sally Morrison-Griffiths, Sue Renwick, Jamie Rylance, Hassan Burhan

**Affiliations:** 1 Liverpool School of Tropical Medicine, Liverpool, UK; 2 Royal Liverpool and Broadgreen University Hospitals, Liverpool, UK; 3 Malawi-Liverpool-Wellcome Trust Clinical Research Programme, Blantyre, Malawi; 4 University Hospital Aintree, Liverpool, UK; 5 Addaction, Liverpool, UK; 6 Liverpool Clinical Commissioning Group, Liverpool, UK

**Keywords:** drug and substance misuse, patient perspectives, heroin, pulmonary disease, chronic obstructive, smoking

## Abstract

**Background:**

Smoking rather than injecting heroin has become more common over the last 20 years. Although there is an increasing body of evidence describing high levels of chronic obstructive pulmonary disease (COPD) in people who smoke heroin, there is limited evidence documenting the impact of the long-term condition on this population group.

**Aim:**

This study aimed to describe the experiences of people who smoke heroin with COPD in Liverpool, UK.

**Design & setting:**

Participants were purposefully sampled for this qualitative study. They included adults enrolled in an opioid replacement clinic run by Addaction in Liverpool, who had already engaged with spirometry testing for COPD as part of a previous study.

**Method:**

Semi-structured interviews were performed with participants with spirometrically confirmed COPD in opioid replacement clinics. Data were analysed using a framework analysis approach.

**Results:**

Sixteen potential participants were invited to take part in the study, of which 10 agreed and were interviewed. Three themes common to all interviews were identified: functional measures of lung health that impacted on their activities of daily living; inhaler and medication perceptions with erratic use that was not concordant with their prescription; and the impact of difficulties accessing care.

**Conclusion:**

These findings, along with previous studies highlighting the prevalence of COPD in this population, warrant efforts to integrate community COPD and opioid replacement services to improve outcomes for this vulnerable population.

## How this fits in

There is a growing body of evidence of high levels of COPD in people who smoke heroin. This appears to happen at an earlier age than the general population. This qualitative study investigates the experiences of these patients and provides clinicians with new insights into the challenges facing this population. These results can be used to help guide the planning of future primary care services for this group of patients.

## Introduction

Over the last two decades, people who use heroin have increasingly smoked rather than injected the drug, understanding this to be a method of harm reduction.^1,2^ This has, however, led many people who smoke heroin to develop COPD at an earlier age than typically seen in those who exclusively smoke tobacco.^3,4^ A recent study in Liverpool demonstrated that approximately 50% of people who smoke heroin have COPD or its overlap syndrome with asthma.^3^ Those with COPD reported extensive respiratory symptoms, such as shortness of breath, cough, and wheeze,^5–7^ which are likely to lead to increased hospital admissions.^8–10^ Screening people who use heroin for COPD may be an important part of providing care early in the course of the disease and limiting the burden of acute hospital care.^4,11^

There is limited evidence regarding patient experience among people who use heroin. The study, therefore, sought insight into the experience and challenges faced by people who smoke heroin with COPD in order to shape future screening and treatment services. This qualitative study examined the lived experience of those with symptoms and a diagnosis of chronic lung disease, and of their experience of interfacing with healthcare services to access treatment.

## Method

Semi-structured interviews were conducted with people who smoke heroin diagnosed with COPD as part of a screening programme between December 2017 and April 2018.

### Study context

The interviews were carried out in Addaction community opioid replacement clinics in Liverpool as part of a COPD screening programme. Addaction is a large independent charity commissioned by the local city council public health department. The screening programme has been previously reported.^3,12^ Between December 2015 and June 2016, participants had been evaluated using respiratory symptom-specific questionnaires, and spirometry to test their lung function. Follow-up screening took place between December 2017 and April 2018.^3,12^

### Recruitment

Within Addaction clinics for opioid replacement therapy, key workers were asked to identify potential participants for the qualitative study. All had been diagnosed with COPD during baseline screening and were current or previous smokers of heroin. Potential participants were given study information prior to their appointment and if they agreed to being interviewed, a key worker who knew them notified the study team. The key worker was not present during the interviews, but was available for support as necessary. Written informed consent was obtained from all participants.

### Data collection

Interviews were led by a researcher (PG) independent of the quantitative screening study, with experience of qualitative research and extensive medical knowledge of COPD. The researcher was aware the participant had COPD but knew no further clinical details. The interviews took place within the two large Addaction clinic sites in Liverpool, in rooms separate from the clinics to remove the participants from the clinical environment, and with refreshments provided. The researcher followed the topic guide, which included: reported symptoms or problems; understanding of COPD; understanding of medications; and experience of access to care. Open questions were asked, with pre-prepared probing questions to gain further information (see Supplementary Appendix S1). All interviews were digitally recorded and identified by participant number only.

### Analysis

A framework analysis approach was used, taking five steps to results: familiarisation; coding; developing and applying a framework; charting; and interpretation.^13^ Each interview was transcribed verbatim, and the transcripts were read through by two researchers (RN and PG) for familiarisation. Following this, RN and PG agreed on the major coded themes based on the topic guide. The data were coded separately in Microsoft Excel, with the transcripts colour-coded for each theme in Microsoft Word. These codes were then shared with the wider research team (RN, PG, JR, HB) and a thematic framework was developed through discussion until a consensus was reached on the mapping of the codes. The themes were shared with Addaction staff members who were part of the research team to ensure they were accurate to the setting and true to the original topic guides (TB and KM).

## Results

Sixteen potential participants were invited, 10 of whom agreed to take part and were interviewed. The age range of those interviewed was 47–59 years, with eight males and two females taking part; all were white. The participants had a range of COPD severity measured by the Global Initiative for Obstructive Lung Disease classifications (mild *n* = 4, moderate *n* = 3, severe *n* = 3).^14^ All were taking opioid replacement therapy, with seven reporting they still smoke heroin. Following the interview framework, three themes common to all interviews were identified: functional measures of lung health that impacted on their activities of daily living; inhaler and medication perceptions with erratic use that was not concordant with their prescription; and the impact of difficulties accessing care.

### Functional measures of lung health

All participants described their COPD in relation to the degree of functional limitation imposed by their symptoms. None of the participants described their COPD or lung disease in terms of medical outcomes such as spirometry results or breathlessness scores. Every participant had concerns about how COPD was affecting their everyday activities of daily life:

*'You know every day I get up in the morning, I go to make a cup of coffee and I come back in and sit down and I’m gasping for breath and I can’t catch my breath, I just can’t get it, you know what I mean?* *'* (Interview 5)

*'I mean I often don’t go out, I struggle carrying shopping, I use one of them now, a bag over your shoulder, with the string, they help me to walk, a lot better. One time, I was putting the bags down, walk a bit, get my breath, again and again, oh God, it was horrendous.* *'* (Interview 10)

Participants described how their functional capacity had deteriorated over time. Eight participants recognised a worsening over a period of months or years. COPD was not explicitly mentioned in these cases, rather the participants described problems with their 'lungs' or 'chest' but did not necessarily attribute their problems to, or label their condition as, COPD:

*'I was just speedy me. I was just one of them people, but now, now I just can’t even, even when my son was 7, I mean he’s 15 now, I was still doing the garden but I couldn’t breathe. I’ve stopped trying to do the garden I mean coz that nearly killed me, I mean it, bad palpitations, I think it would have killed me that day, my neighbour came out and said "go in, get in now!" and stayed with me and everything.'* (Interview 9)

*'I can’t any more, I was quite active say 10 years ago, but now I can’t run, can go slow on a bike but that’s it, couldn’t run if I wanted to ... yeah, just from walking, or as I’ve said just getting out of bed, I can walk to the landing to look out the window and be standing panting, thinking "where’s the inhalers" and it’s only when I have the inhalers that it seems to calm me down a bit.* *'* (Interview 3)

Two participants did not perceive their chest to be a significant problem; both described functional limitation, but reported it as normal for them:

*'I did run for the bus the other day and feel a bit out of breath, but most people would wouldn’t they? Running for the bus and exerting themselves, you know, at 8 o’clock in the morning … I’ve got to be very fit and active every single day … So my chest isn’t too bad considering what I’ve put into it over the years, at least from my perspective it’s not.* *'* (Interview 2)

*'No … no it doesn’t stop me, and the inhalers I don’t use them every day, only when I need to, only when I’m coughing and short of breath I’ll use them then … yeah but only when I’m coughing, sometimes I cough and I’m nearly puking ... maybe two or three times a week*.*'* (Interview 4)

### Inhalers and medication perceptions

All participants reported taking at least one inhaler and talked about them consistently during the interviews. Of the 10 participants interviewed, seven reported that they had recently borrowed other people’s inhalers or medication to help their chest. The reasons for this were not always identifiable, but access to medication was a frequent difficulty:

*'... well the last one I needed I got off me cousin coz she had a spare.'* (Interview 4)

*'My girlfriend has one of those nebuliser things so I just throw myself on that, and erm, that kind of makes me feel ok.* *'* (Interview 6)

*'If there’s antibiotics in the house then I’ll use them instead* [of going to a doctor] *… it’s like, I’ve been around drugs most of my life, so I’m not afraid to try an antibiotic or something if I think it might help me … well the wife has got asthma there, so err she got inhalers, a brown one and a blue one, and I’ll use them every now and again … They’ve told me COPD is different to asthma so instead of using the wife’s stuff, I might need something else, a different one for my condition might be better.* *'* (Interview 7)

Participants also reported the use of metered-dose inhalers (MDIs) as drug pipes, either by themselves or others:

*'We used to make bongs out them, the blue one* [salbutamol MDI]*, years ago, but God I couldn’t even look at doing that now it knocks me sick.'* (Interview 9)

*'You just* [describes process of converting inhaler into a drug pipe] *... Yeah yeah the blue ones, the hollow tube* […] *I’ve seen a lot of people use them like that, like "rock pipes" we called them ... So it’s a common thing to do ... It sounds mad that people are using things meant to help you breathe for that stuff doesn’t it?'* (Interview 4)

There was generally poor knowledge and understanding surrounding inhalers, with participants confused between the name of the inhaler and the colour; for example, describing a 'blue inhaler' but pointing to a purple (Seretide) inhaler (interview 1). In general, participants described high usage of the 'blue inhaler' (salbutamol) and inconsistent use of long-acting medication such as tiotropium or formoterol. Participants reported that they used inhalers as and when they needed them, and there appeared to be a lack of understanding of using longer-term inhalers as prevention for some of the functional problems they described:

*'I use the inhalers carefully, don’t waste them you know, use them as and when I feel I really need them, I don’t use them for the fun of it, I’ll only use them when I feel as though I’m struggling.'* (Interview 8)

*'Talking about the “blue and pink”? Nah I don’t use any of them regularly, only when I get bouts of it.'* (Interview 4)

### Impact of access to care

The difficulty in accessing both primary care appointments and hospital appointments was a recurring theme. The participants described travel to and from appointments as a barrier to attending both the GP surgery and the hospital. In some circumstances the cost of travel was prohibitive and in others it was the practicalities of getting out of the house while being unwell. The participants suggested that they may attend more often if access to care was easier:

*'... well it’s two buses away, and it kills me to get there, but I love that doctor so I make the effort if I need to.'* (Interview 1)

*'It can be a bit of a task though, with the breathing and that, sometimes I only have to walk around the corner and I’m having a bad time ... yeah well I have to walk here as well so that can be hard in itself, stop and rest about 20 times ... you’d see me sat down on the road and all that, you know what I mean?'* (Interview 6)

*'I just can’t get there, even if I phone a taxi to come and take me, I just can’t get up and down the stairs.'* (Interview 8)

The experience that participants had of their GPs and hospital doctors varied throughout the interviews, with participants describing their GP as *'* *marvellous* *'* (interview 1) and saying *'* *I can’t fault him* *'* (interview 10). All participants reported that seeing their own GP was difficult; either they saw a locum, which they reported as a negative experience, or that getting an appointment via reception was difficult:

*'... well it’s hard to get an appointment at the doctors, to get my script, and God did I feel it when I ran out, really bad.'* (Interview 1)

*'... you know the credit on your phone to ring up, and you know when they say to ring at 8 o’clock in the morning, well try using somebody else’s phone, like I’ve had to use before, at eight in the morning. That’s no good to me.'* (Interview 10)

*'... maybe receptionists, when you phone up you’ve got to phone at certain times, waiting to get through to the receptionists, you see it’s like you wait for the phones to turn over at 8:20am and you might not get through ‘til 9:20am and they’ve all gone, so, that’s not good ... like I could have a problem like today, and I’ll go in or ring up and they’ll say "yeah we can fit you in in 2 weeks’ time", well hang on, you know?*’ (Interview 8)

The participants generally reported that they did not attend hospital appointments. There were a variety of reasons for this, including the feeling of stigma or that chest problems were ‘self-inflicted’. Several participants avoided secondary care environments owing to negative associations with, for example, a dying relative, or a negative experience with the hospital staff:

*'I think they’re rushed off their feet, so you know, I think there’s sicker people than me to be seen to … coz mine are self-inflicted, like they see sick children and it just shows you that I’ve done this to myself, you know? ... because it’s self-inflicted isn’t it? I just feel embarrassed wasting the national health money ... '* (Interview 1)

*'... well they can’t do nothing can they, the doctors can’t do nothing for my chest the way it is, I have inhalers, I asked them about nebulisers, they said I don’t need a nebuliser, so, but I think I do like ... I don’t know, I feel like they just look at you and think "ah its self-inflicted" and that ...* *'* (Interview 5)

*'* [when admitted with chest problem] I’m on certain medication that they just won’t give you, like the methadone, they just won’t give you that, so I’ve got to lie there, like if I go in on a Friday I’ve got to lie there until Monday, with no nothin’. So you’re in bits by Monday, they sort your dose out, I know people might try and cheat the system, but you know, it goes on, I’m not going to abuse it and as doctors and nurses you’ve got to think "is this man on this?". For one time in the hospital I was on 360 ml a day, so when the doctors read that they thought "F*ck off no way", you’d say as well. I mean I’m not on that now. They say to you get a stat dose of 20 ml, but what that’s gonna do for me when I’m on 360? It doesn’t help. It’s experiences like that, the last thing I’d want to do is ring an ambulance if I can help it.' (Interview 8)

## Discussion

### Summary

Using qualitative interviews, this study has shown the key challenges for this cohort are lung health symptoms that impacted on their activities of daily living; inhaler and medication perceptions, with erratic use that was not concordant with their prescription; and the impacts of difficulties accessing care. These themes occurred throughout the interviews and could help inform and develop respiratory services for this group of patients.

### Strengths and limitations

The main limitation of this study is the small number of interviews conducted and the breadth of the interviews conducted. It may have added to the depth of knowledge if healthcare providers had also been interviewed, and further study in this area would be informative. The participants who declined to take part may have led to bias, with those happier to engage or those with strong opinions about their COPD potentially more likely to take part. This study has, however, offered a new and unique view on the possible barriers facing people with COPD who smoke heroin.

### Comparison with existing literature

The participants’ main focus was the functional limitation that they experienced in their activities of daily living as a result of their COPD. No participant discussed the details of their medical diagnosis, the staging of their COPD or used medical scoring, such as MRC (Medical Research Council) and COPD assessment test (CAT), to describe their COPD. To the best of the authors' knowledge, there are no studies that specifically describe the impact and functional limitations of COPD in people who smoke heroin; however, the themes identified in this study are similar to studies of people who smoke tobacco. In a large pan-European study examining patients with COPD and their experience of acute exacerbations, there was wide variability in reported symptoms between patients (reported at 62.7%), high levels of self-medication, and poor understanding of their condition.^14^ The impact of COPD-related symptoms on activities of daily living has been evaluated previously, with there being a high prevalence of impaired functioning among patients with COPD across a wide range of domains. The impairment does not clearly correlate with standard clinical measures such as degree of airflow obstruction or level of dyspnoea.^15,16^ Furthermore, functional limitation was common among the participants in the study and was often deemed to be ‘normal’ by individuals. Relying on objective measures of COPD severity in this patient population may not identify individuals who would benefit from more targeted interventions, such as pulmonary rehabilitation, aiming to improve symptoms and level of functioning with regards to activities of daily living.

In general, the study participants appeared to be taking COPD medication in an irregular and often self-directed manner. In some, inhalers were used as a vehicle for smoking drugs rather than for their intended purpose. The engagement with the primary care system in the UK was mixed, with some participants having an excellent experience of their GP, while others found significant barriers to attending primary care, with travel and access to the GP being the principle negative factors. Participants universally found accessing hospital treatment difficult, with stigma and a feeling of having a 'self-inflicted' illness limiting attendance at the hospital.

Poor adherence and a lack of knowledge or trust in medication has been reported in other studies of COPD patients, with overuse, underuse, and alteration from medication schedule commonplace.^17^ In a cross-sectional study of 173 patients with COPD attending outpatient clinics, 29.5% of attendees had 'low adherence' to medication.^18^ The theme of accessing prescription medication from peers or family members is also not unique to the study population, with another study on students finding that those who access medication from peers were also more likely to use illicit drugs.^19^ Stigma and significant barriers to accessing both primary and secondary care are common across illicit drug users, with reports of 'dissonant care' commonplace with other qualitative and narrative studies in this population, describing similar findings to the analysis of the interviews in the present study.^20–23^

### Implications for research and practice

The data highlight important considerations in the development of COPD services for drugs users. Clinicians should consider functional outcomes as well as clinical outcomes and objective scoring systems when discussing COPD treatment with patients. There is potential that functional goals would be welcomed by this patient group and may help motivate the attendance to care and adherence to treatment. The use of self-medicated inhalers, nebulisers, and other drugs makes clinical assessment even more challenging in this population. It is highly likely that the standard methods of assessing 'medication pick-up' at pharmacies does not provide an accurate picture, and that self-reported usage may be key to determining the real clinical need. Alongside this, the overuse of some inhalers, potentially as drug paraphernalia, adds to an already complex clinical picture. The access to respiratory care for this patient group is limited, with barriers including transport to hospitals and a feeling of stigma. There is potential that providing respiratory care centred around venues where patients attend opioid replacement therapy would improve continuity of care and assist in obtaining accurate medication histories. This research highlights the complex needs of people who smoke heroin with COPD and the need to consider their functional limitations, medication management, and access to care in future planning of respiratory services.
